# Extracellular Vesicles in Liquid Biopsies: Potential for Disease Diagnosis

**DOI:** 10.1155/2021/6611244

**Published:** 2021-01-11

**Authors:** Jialing Liu, Ye Chen, Fang Pei, Chongmai Zeng, Yang Yao, Wen Liao, Zhihe Zhao

**Affiliations:** ^1^Department of Orthodontics, West China Hospital of Stomatology, State Key Laboratory of Oral Diseases & National Clinical Research Center for Oral Diseases, Sichuan University, Chengdu, China; ^2^Department of Implantology, West China Hospital of Stomatology, State Key Laboratory of Oral Diseases & National Clinical Research Center for Oral Diseases, Sichuan University, Chengdu, China

## Abstract

Liquid biopsy is conducted through minimally invasive or noninvasive procedures, and the resulting material can be subjected to genomic, proteomic, and lipidomic analyses for early diagnosis of cancers and other diseases. Extracellular vesicles (EVs), one kind of promising tool for liquid biopsy, are nanosized bilayer particles that are secreted by all kinds of cells and that carry cargoes such as lipids, proteins, and nucleic acids, protecting them from enzymatic degradation in the extracellular environment. In this review, we provide a comprehensive introduction to the properties and applications of EVs, including their biogenesis, contents, sample collection, isolation, and applications in diagnostics based on liquid biopsy.

## 1. Introduction

Liquid biopsy is conducted through minimally invasive or noninvasive procedures, the samples are simple to store, and they can be processed fast enough to provide real-time information. The term “liquid biopsy” is derived from the term “tissue biopsy.” Tissue biopsies, in contrast, are quite invasive, involving the risk of complications, and cannot provide real-time information [1]. In addition, the insights from tissue biopsies may be biased because they reflect the state of only one part of the tissue. Liquid biopsies contain numerous potential cells or particles that could be analyzed: extracellular vesicles (EVs), circulating tumor DNA, circulating tumor cells, circulating endothelial cells, and cell-free fetal DNA [2]. Among these components of liquid biopsies, EVs have attracted researchers' interest because they have advantages over other analytes, such as stability in the circulation.

**Figure 1:**
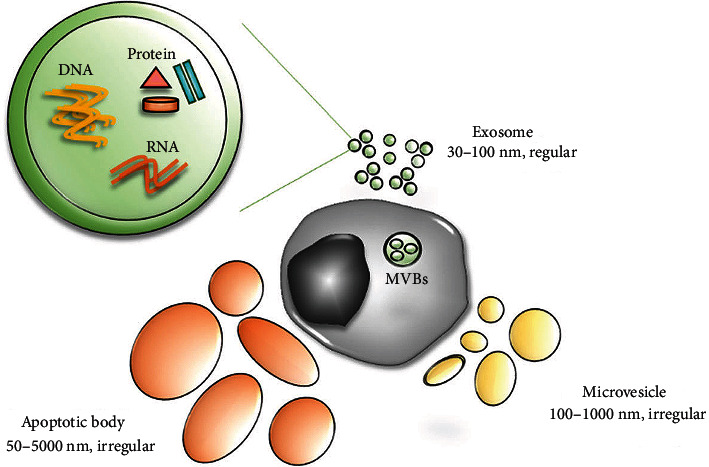
Three main classes of extracellular vesicles: microvesicles, exosomes, and apoptotic bodies. Reprinted from Kim et al. [195].

EVs are lipid bilayer-enclosed particles released from all types of cells and found in biological fluids such as blood, cerebrospinal fluid (CSF), urine, saliva, breast milk, seminal fluid, and tears [3, 4]. EVs were first reported in 1946 by Chargaff and West after they ultracentrifuged blood plasma and obtained particles with procoagulant properties [5]. In 1967, Wolf reported that this coagulant material in high-speed supernatants originated from platelets and named it “platelet dust” [6]. This “dust” did not attract much attention until the 21st century after EVs were identified as potential vehicles to transfer signaling molecules from cell to cell. Since then, research has revealed three main classes of EVs: microvesicles, exosomes, and apoptotic bodies [7]. Microvesicles are directly created by outward budding of the plasma membrane (PM), while exosomes originate from intraluminal vesicles produced by inward budding [8]. Apoptotic bodies arise when cells undergo apoptosis, and they are not covered in the present review (Figure 1). EVs are an attractive liquid biopsy tool as they contain proteins, lipids, and nucleic acids from their parental cells, which may be tumor cells or other types of diseased cells, and they can sensitively reflect an individual's health status [9, 10].

It is worth pointing out that membranous EVs and molecules entrapped and enclosed in EVs show good stability in both morphology and chemical property. The lipid bilayer surrounding EVs protects the biocargo from extracellular proteases and other enzymes. For example, one study suggested that phosphoproteins could be recovered from EVs isolated from plasma that had remained frozen longer than five years [11]. Similarly, another study found that storing EVs at 20°C or subjecting them to multiple rounds of ultracentrifugation did not substantially alter their size [12]. Luminal protein TSG101 has been shown to remain quite stable within EVs [13], so do DNA [14], microRNAs (miRNAs) [15], and circular RNAs (circRNAs) [16]. The stability of EVs and their contents makes them promising biomarkers.

In this review, we summarize the biogenesis and contents of EVs as well as their isolation techniques from biological fluids. From our point of view, EVs are promising tools for liquid biopsy, especially for diagnoses based on the proteins, nucleic acids, and lipids within the EVs.

## 2. Biogenesis and Contents of EVs

### 2.1. Biogenesis of EVs

All cells are able to release EVs, including exosomes, into the extracellular space [17]. The biogenesis of exosomes is as follows. First, the PM invaginates to produce a cup-shaped structure containing fluid, lipids, proteins, metabolites from the extracellular milieu, and cell surface proteins. This inward budding or endocytosis generates early-sorting endosomes, which mature into late-sorting endosomes. Next, intraluminal vesicles are generated and accumulate in late-sorting endosomes. Cytoplasmic constituents enter the intraluminal vesicles and ultimately become the cargo of the future exosomes. Late-sorting endosomes containing intraluminal vesicles give rise to multivesicular bodies. In most cells, multivesicular bodies fuse with autophagosomes or lysosomes, and the contents are ultimately degraded by lysosomal hydrolases. However, multivesicular bodies bearing markers such as lysosome-associated membrane proteins LAMP1/LAMP2, the tetraspanin CD63, or other molecules can be transported to the PM, where they release their contents into the extracellular milieu [17, 18]. In this way, exosomes encapsulate substances from the parental cells.

This process of exosome biogenesis is regulated by mechanisms dependent on endosomal sorting complexes required for transport (ESCRT), as well as by ESCRT-independent mechanisms. The ESCRT machinery is an evolutionarily conserved membrane remodeling complex that participates in a broad range of physiological and pathophysiological processes such as biogenesis of exosomes and release of envelope retroviruses [19]. The ESCRT machinery is composed of nearly 30 proteins that assemble into four complexes. Among them, ESCRT-0, ESCRT-I, and ESCRT-II are stable polymers, whereas ESCRT-III forms through the dynamic assembly of monomeric proteins [20]. The ESCRT-0 complex, which can recognize and cluster ubiquitinated transmembrane proteins, is a heterodimer made up of signaling transducing adaptor molecule (STAM) and hepatocyte growth factor-regulated tyrosine kinase substrate (HRS). ESCRT-I is instrumental for inducing membrane budding and sorting cargoes into multivesicular bodies. ESCRT-III is dominant in driving membrane scission. ESCRT-I and ESCRT-III are connected by ESCRT-II, which is a heterotetramer consisting of four vacuolar protein sorting- (VPS-) associated proteins (one VPS22, one VPS36, and two VPS25) [21, 22].

Exosomes can also be formed in the absence of ESCRT complexes, which is called the ESCRT-independent mechanism. In experiments involving mouse oligodendroglial cells that can secrete proteolipid protein- (PLP-) containing exosomes, knockdown of HRS, Alix, or TSG101 to block the generation of ESCRT machinery components did not affect inward budding of PLP [23]. Budding was, however, blocked by the neutral sphingomyelinase inhibitor, which inhibits the formation of ceramide in exosomes. These results led to the discovery of ceramide-triggered budding of exosome-associated domains into multivesicular endosomes. Several membrane proteins of the tetraspanin family, such as CD63, CD9, CD81, and CD82, have been implicated in the sorting of cargoes and the formation of exosomes [24].

**Figure 2:**
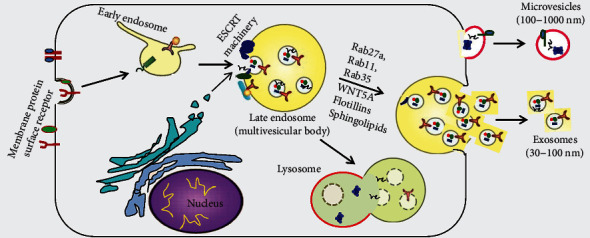
Schematic depiction of biogenesis of exosomes and microvesicles, two types of extracellular vesicles. Reprinted from Ailawadi et al. [196].

In contrast to exosomes, microvesicles form through outward budding from certain microdomains in the PM, especially microdomains enriched in cholesterol and sphingolipids [25]. Changes in the Ca^2+^ level and in the composition of lipid and protein within the PM have been implicated in the shedding of microvesicles into the extracellular space. These changes are driven by enzyme machines, including scramblases, calpain, and aminophospholipid translocases such as flippases and floppases, which favor the flopping of phosphatidylserine (PS) from the interior to exterior membrane leaflets [26]. The flopping of PS results in bending of the PM, restructuring of the underlying cytoskeleton, and biogenesis of microvesicles. However, microvesicle biogenesis can also proceed without the flopping of PS, which suggests that other lipids also contribute to the budding of microvesicles [27]. The PM and cytoskeleton are tightly connected, and cytoskeletal changes are associated with the biogenesis of microvesicles. Thus, cytoskeletal elements and their regulators are considered to contribute to microvesicle biogenesis [28]. Actin is perhaps one of the best-studied cytoskeletal elements. *β*- and *γ*-cytoplasmic actins facilitate endothelial microvesicle formation [29]. The ras homolog family member A (Rho A) is a classical Rho GTPase that regulates cytoskeleton function, especially actin stress fiber formation. It is not surprising, then, that a Rho A-associated signaling pathway has been implicated in the generation of microvesicles (Figure 2) [30]. The biogenesis of EVs is quite complex and is still being elucidated, and scientists are still on the way of exploration.

### 2.2. Biomolecules Contained in EVs

EVs contain heterogeneous contents from their cells of origin, including proteins, miRNAs, messenger RNAs (mRNAs), circRNAs, DNA, and lipids, potentially offering a complete range of biomolecules for assessing the original cell's health or disease condition.

**Figure 3:**
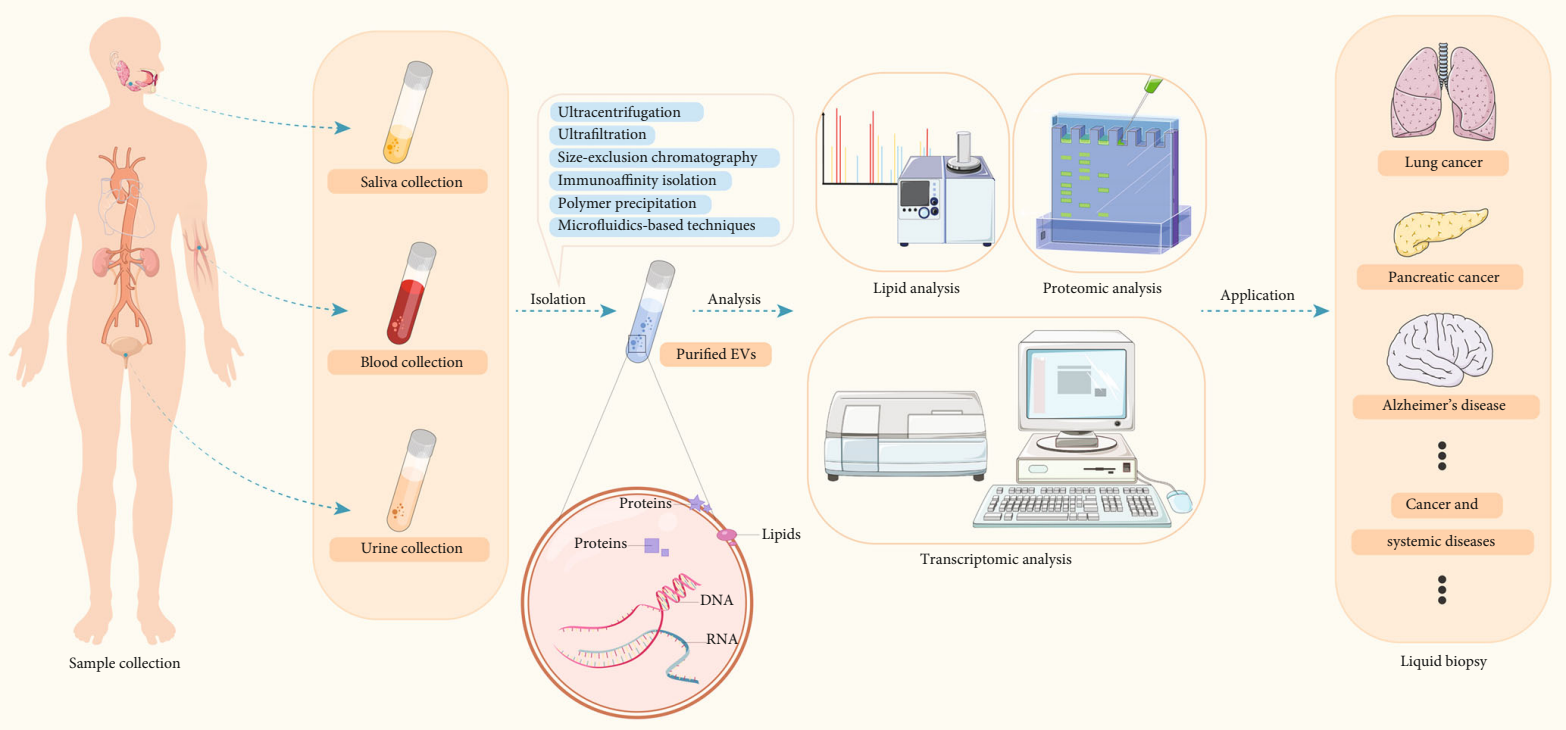
Different methods to isolate extracellular vesicles. Reprinted from Pang et al. [93].

The range of the EV biocargo so far reported in the literature has been cataloged in EVpedia (http://evpedia.info), Vesiclepedia (http://www.microvesicles.org), and ExoCarta (http://www.exocarta.org) [31–33]. A range of analytical techniques can extract valuable information from the heterogeneous EV biocargo (Figure 3). These techniques include SDS-PAGE, western blotting, enzyme-linked immunosorbent assay, flow cytometry, microfluidic assays, and mass spectrometry- (MS-) based proteomic analysis, all of which are used for protein analysis; nucleic acid sequencing, which is applied for nucleic acid analysis; and liquid chromatography and gas chromatography coupled to MS (LC-MS and GC-MS), which are able to analyze lipid species in EVs [34–36].

#### 2.2.1. Proteins

Thousands of proteins, cytosolic and membrane-bound, have been found in EVs. Proteins traditionally used to identify EVs are CD9, CD63, CD81, annexins, ESCRT proteins, and TSG101 [37], all of which participate in vesicle sorting and trafficking. Proteins in EVs have attracted substantial attention as disease biomarkers. Assaying tumor-derived proteins directly from body fluids lacks sensitivity because the proteins have been diluted and degraded, but assaying proteins in tumor-derived EVs can work much better because the proteins are locally concentrated and protected within the vesicles [38]. For example, programmed death-ligand 1 (PD-L1) protein in exosomes may be a biomarker for metastatic melanoma, non-small-cell lung cancer, and head and neck squamous cell carcinoma [39–41]. Glypican-1 in exosomes may be a biomarker for pancreatic, colorectal, and breast cancers [42–44]. EV-resident diagnostic biomarker proteins may include DEL-1 [45], MIF [46], and CEA [47].

#### 2.2.2. Nucleic Acids

In 2007, Valadi and coworkers discovered that exosomes contain mRNAs and miRNAs that can be transferred to recipient cells, where they can function [48]. Intriguingly, many exosomal miRNAs and mRNAs are not detectable in body fluids, suggesting that they may be highly specific noninvasive biomarkers. For example, small RNA sequencing of exosomes from the plasma of patients with papillary thyroid cancer identified miR-485-3p and miR-4433a-5p as potential diagnostic biomarkers [49]. Levels of exosomal miR-485-3p differed between patients at low or high risk of illness. Levels of the mRNA encoding chemokine (C-C motif) ligand (CCL2) in urinary exosomes may reflect the severity of IgA nephropathy [50], which is the most common primary glomerular disease and the primary driver of end-stage kidney disease [51, 52].

Before 2014, EVs had been reported to contain only ssDNA and mitochondrial DNA. Subsequently, they were also found to contain dsDNA; in fact, they were found to contain material from all chromosomes, potentially providing an extremely detailed picture of the genetic makeup of the parental cells, including the presence of mutations [53, 54]. For example, DNA analysis of exosomes from cultures of non-small-cell lung cancer cells has detected mutations in epidermal growth factor receptor [54], and DNA analysis of exosomes from the serum of pancreatic cancer patients has detected mutations in KRAS and p53 [53].

#### 2.2.3. Lipids

Compared with their parental cells, which are enriched in phosphatidylcholine, EVs are generally enriched in phosphatidylserine, cholesterol, sphingomyelin, and glycosphingolipids. The ratios of EVs versus those of parental cells are about 2.1 (sphingomyelin and phosphatidylserine) and 1.4 (cholesterol) [55]. The lipid composition of EVs can be studied in detail using mass spectrometry, high-performance liquid chromatography, nuclear magnetic resonance, gas chromatography-mass spectrometry, and computational approaches [56–62]. One study identified differences in lipid composition between EVs of ovarian cancer cells and ovarian epithelial cells, and these differences may facilitate early diagnosis [63]. Another study found that analysis of exosomal lipids was able to distinguish non-small-cell lung cancer in the early or late stages [64]. A third study identified nine lipid species, including PS and lactosylceramide, whose levels differed significantly between prostate cancer patients and healthy controls [65].

## 3. Sample Collection before Isolation of EVs

EVs are intercellular shuttles released by most cells, and they can be found in nearly all body fluids [66]. Different body fluids have different characteristics, such as density and viscosity, as well as different compositions. For example, blood is denser and more viscous than urine. Urine contains creatinine, urea, and uric acid, while saliva contains amylase and lipase. Therefore, these different fluids should be collected and processed in different ways in order to isolate EVs. Three kinds of body fluids commonly used for harvesting EVs are described.

### 3.1. Blood (Plasma/Serum)

Plasma and serum are blood cell-free fractions obtained through centrifugation. A global survey of members of the International Society for Extracellular Vesicles indicated that 47% of respondents isolated their EVs from plasma and 22% from serum [67]. The efficiency of EV isolation can depend on numerous factors linked to the quality of the blood sample, including donor age, medical history, diet, time of sample collection, choice of anticoagulant, and venipuncture [68–70]. In fact, the physical forces during blood drawing can activate platelets and trigger the release of platelet-derived EVs, altering the quality of the blood samples. Therefore, venipuncture should preferably be conducted from the same site using a nonbutterfly needle of gauge 21 or larger [71, 72]. The most suitable anticoagulant depends on the downstream analyses to be conducted. For instance, heparin is not recommended if the samples will subsequently be analyzed using PCR because it can competitively inhibit the binding of primers and enzymes to the nucleic acid template [73]. Alternatives include citrate-theophylline-adenosine-dipyridamole (CTAD), citrate, ethylenediaminetetraacetic acid, and sodium fluoride/potassium oxalate. CTAD inhibits platelet activation [74], and adding it to EDTA can reduce platelet clumping [75]. In 2012, the International Society on Thrombosis and Haemostasis recommended citrate as the anticoagulant for EV studies, and it is currently in wide use [76].

### 3.2. Urine

Urine is a metabolic by-product flowing from the kidney to the bladder. Urinary EVs are quite stable, and their biomolecular cargo is protected from the ribonucleases, proteases, and lipases in urine [77]. Sampling urine is less invasive than sampling blood, and the miRNA content of urinary EVs correlates with that of serum EVs [78]. Therefore, urine is extremely attractive as a source of EVs. Isolating intact EVs from urine is a challenge because of the high concentration of Tamm-Horsfall protein (THP), also known as uromodulin, which is the most abundant glycoprotein in urine [79]. THP can trap EVs to form filamentous networks, interfering with EV isolation. Adding dithiothreitol can reduce disulfide bonds linking the THP monomers, not only releasing the entrapped EVs but also potentially altering the structure of proteins of interest [80]. The detergent 3-[(3-cholamidopropyl)dimethylammonio]-1-propanesulfonate (CHAPS) may be superior to dithiothreitol because it can reduce THP interference without altering EV morphology or exosomal marker distribution, but the method is time-consuming [81]. In another lengthy procedure, raising the pH and reducing ionic strength can also disrupt the association between THP and EVs [82]. A faster method that can preserve the structure of proteins of interest may be hydrostatic filtration dialysis, combined with urea denaturation and depolymerization of THP [79].

### 3.3. Saliva

Saliva is a physiological fluid produced and secreted by salivary glands, comprising minor salivary glands and three major glands (submandibular, parotid, and sublingual). It plays a vital role in lubrication, digestion, and mastication, and it is the first line of defense against pathogens, as it contains various immunoglobulins and enzymes. A healthy individual secretes 600 ml of saliva per day, so obtaining adequate saliva samples is usually straightforward. The quality of EVs isolated from saliva can depend on numerous factors that affect the quality of the saliva sample, including the timing and location of sampling [70, 83, 84], as well as the technique of sample collection [85], which can be through passive drooling [86] or stimulation by chewing or administration of a chemical [87]. Concentrations of salivary tissue factors [88] and cortisol [89] follow a circadian rhythm, which can affect the isolation and analysis of EVs. It may be better to collect saliva only from the parotid gland [90]. Eating, drinking, smoking, and exercising can affect saliva content [91], so individuals should smoke and drink in moderation as well as avoid exercise before sample collection. They should refrain from eating for one hour before collection.

## 4. Isolation of EVs

**Figure 4:**
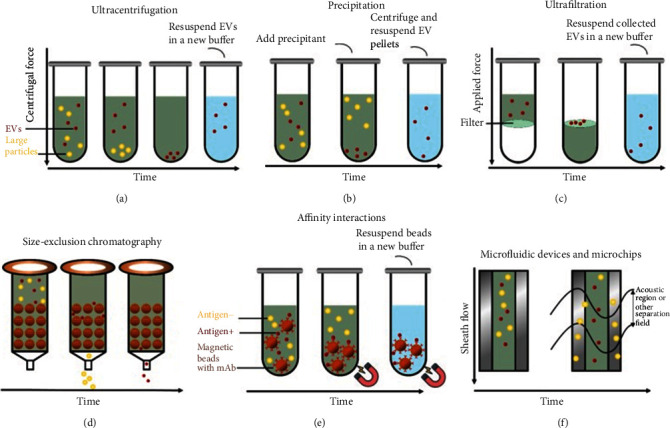
Schematic representation of the analysis of extracellular vesicles in liquid biopsies for clinical medicine.

Many studies have examined the diagnostic potential of EVs, but their clinical application is limited by the lack of simple, efficient procedures to obtain EVs with high purity. Six major isolation strategies have been published, each with its own advantages and limitations, including ultracentrifugation, polymer precipitation, ultrafiltration, size-exclusion chromatography, affinity isolation, and microfluidics-based techniques (Figure 4) [92, 93].

### 4.1. Ultracentrifugation

Ultracentrifugation methods, namely, differential ultracentrifugation or density gradient centrifugation, are usually applied to isolate EVs from biofluids [70] (Figure 4(a)). Differential ultracentrifugation is regarded as the gold standard for EV isolation [67] and is based on the fact that the centrifugal force pulls larger and denser particles into the pellet [94]. This isolation method, although relatively simple and cheap, leads to EVs that can be contaminated with protein aggregates, especially when the starting sample is serum or plasma, and it requires large initial volumes [95].

A more effective variation of this method is density gradient ultracentrifugation, which involves ultracentrifugation through a density gradient. This method can lead to higher EV yield and lower protein contamination than conventional differential ultracentrifugation [95]. Similar to differential ultracentrifugation, the principle of density gradient centrifugation is also based on the size, shape, mass, and density of EVs. As an example of applying this method, the body fluid sample is placed at the top of a density gradient with density decreasing from the bottom to the top in a centrifuge tube. The density gradient is typically iodixanol or sucrose. The application of a centrifugal force causes the solutes in the sample to move through the gradient at a characteristic sedimentation rate, allowing different components to separate from one another. Density gradient ultracentrifugation is increasingly popular as it leads to higher EV yield and lower protein contamination than differential ultracentrifugation [95]. The combination of two types of density gradient ultracentrifugation, namely, coupling rate-zonal centrifugation or isopycnic-zonal centrifugation, with differential ultracentrifugation, may lead to EVs of higher purity [96]. The drawback of this combination method is it requires additional preparation, time, and cost.

### 4.2. Size-Based Techniques

Isolation of EVs can be performed using ultrafiltration, which is a size-based isolation technique consisting of semipermeable membrane filtration (Figure 4(c)). This separation process is usually used for purifying and concentrating protein solutions, and later, it was found to be effective in isolating EVs. Larger particles such as EVs are retained by the filter, while smaller particles pass through [97]. This approach is faster and easier than ultracentrifugation, and a relatively small sample can provide adequate material, such as 0.5 ml urine [98]. However, “cakes” can form on the filter and block it, and EVs can deform at the filter interface as a result of pulling forces. To solve this problem, in 2018, Busatto et al. invented a novel size-based filtration method named tangential flow filtration [99], which is gentler and can avoid filter clogging, resulting in higher yield. Commercially available kits have been developed that rely on size-based isolation. In these kits, the sample is forced through two membranes, a 200 nm membrane at the top and a 20 nm membrane at the bottom. The lower membrane captures EVs < 200 nm or >20 nm, while larger vesicles are retained on the upper filter, and the smallest vesicles are discarded [100]. Size-exclusion chromatography also isolates EVs by size: smaller molecules slow down because they enter the pores of the gel, while EVs do not enter the pores and flow through faster [101] (Figure 4(d)). This technique can preserve EV structure better than ultracentrifugation and ultrafiltration. However, it cannot effectively separate EVs from similarly sized lipoproteins or protein aggregates.

### 4.3. Immunoaffinity Isolation and Other Methods

Immunoaffinity isolation is based on the presence of surface proteins or antigens on the EV membrane: antibodies are used to bind these antigens and thereby isolate the desired EV subpopulation (Figure 4(e)). The antibodies can be attached to magnetic beads, culture dishes, resins, and other substrates. For instance, melanoma-derived exosomes have been captured from plasma using magnetic beads carrying the anti-CSPG4 monoclonal antibody [102]. Many proteins have been explored as biomarkers for capturing EVs, including CD63, CD34, and CD326. Some researchers have suggested that CD63 lacks adequate specificity, leading them to develop an immunoaffinity-based microfluidic isolation device called the ^new^ExoChip with higher specificity of EV isolation [103]. An enzyme-linked immunosorbent assay has also been introduced to isolate EVs, in which the antibody is immobilized on a microplate [104]. This method can isolate EVs from plasma, serum, and urine samples as small as 100 *μ*l. While immunoaffinity capture allows the isolation of specific EV subsets, current methods cannot exploit intracellular antigens. Another problem is that the EVs eluted from magnetic beads can lose some of their activity. Immunoaffinity methods are expensive, and yields of purified EVs are low. There is another isolation method called polymer precipitation, whose principle is that a hydrophilic polymer or reagent is added to the sample, and the polymer interacts with water surrounding the EVs, causing them to precipitate (Figure 4(b)). This method is rapid and provides a high yield [105], but EV purity can be low because the polymer precipitates not only EVs but also any water-soluble material, including lipoproteins and nucleic acids [106, 107].

Although the above-listed methods could be applied for EV isolation, only microfluidics-based techniques can combine EV isolation and disease detection in one platform (Figure 4(f)). They offer portability, fast isolation, cost-efficiency, and small starting volume. Size-based microfluidics use nanowire and micropillar structures to separate EVs with diameters in a certain range from smaller cellular debris, proteins, and other particles [108]. This technique can isolate various subtypes of EVs and minimize contamination by proteins and other nanoparticles, but it requires complicated photolithography fabrication, saturation limits are relatively low, and recovery is slow. Immunoaffinity-based microfluidics separates EVs through the interaction of surface or intravesicular EV biomarkers with antibodies immobilized onto the microchannel surface or magnetic beads. Dynamic exosome microfluidics utilizes an electrical or acoustic field to separate EVs from other nanoparticles [109]. By changing field magnitude and frequency, this technique can separate EV subtypes without the need for complicated photolithography fabrication.

## 5. Diagnosis of Disease Based on Analysis of EVs in Liquid Biopsies

Exploring diagnostic methods based on the analysis of contents in biofluids has become a hot research topic in recent years. Because EVs are stable and carry diverse cargo molecules, they are considered a promising tool for noninvasive diagnosis. Most studies of liquid biopsy have focused on cancer, especially lung cancer. The first important milestone in liquid biopsy came in 2016 when the US Food and Drug Administration approved the first diagnostic test for lung cancer based on circulating tumor DNA in blood samples [110]. Studies have also examined EVs in other diseases, such as severe interstitial fibrosis and tubular atrophy in postrenal transplantation [111].

Plasma and serum samples are the most frequent forms of liquid biopsies from which EVs are purified, but EVs isolated from urine and saliva can also be clinically useful (Figure 3). Contents of EVs can depend on the biofluids from which they were isolated [78]. The differences of RNA profiles of EVs isolated from serum and urine of patients with cholangiocarcinoma were reported, indicating that for certain diseases, EVs purified from urine may provide differential diagnostic accuracy compared with EVs from blood [112]. In patients with diabetic kidney disease, their miRNAs from urinary and serum EVs show moderate to strong correlations with each other [78], but further work is needed to determine whether one type of sample is better for clinical analyses. In patients with lung cancer, the proteomes of exosomes were found to differ between saliva and serum [113]. Based on the fact that EVs isolated from specific biofluids may provide specific diagnostic information for certain diseases, we described diseases according to the classification of biofluids (blood, urine, or saliva).  Table 1 summarizes typical examples of EV contents that may serve as biomarkers.

### 5.1. Blood (Plasma/Serum)

#### 5.1.1. Blood-Based Liquid Biopsy in Cancer

Non-small-cell lung cancer, breast cancer, pancreatic cancer, colorectal cancer, ovarian cancer, and nasopharyngeal carcinoma can be detected on the basis of blood-derived EVs. Non-small-cell lung cancer, a subtype of lung cancer, is the leading cause of cancer-associated mortality worldwide [114]. Proteins and miRNAs within blood-derived EVs can aid in diagnosis; such proteins include epidermal growth factor receptor, NY-ESO-1, PLAP, EpCAM, and Alix [115], as well as fibronectin [116]. In addition, levels of alpha-2-HS glycoprotein and extracellular matrix protein 1 in serum EVs are significantly higher in patients than in healthy controls, suggesting diagnostic potential [117]. Potentially diagnostic EV-derived nucleic acids include the long noncoding RNA called growth arrest-specific transcript 5, whose levels in serum EVs are significantly lower in patients than in healthy controls [118]. It has been suggested that a *single biomarker* detection is not fully adequate, and combining miRNA and protein markers may be particularly effective at diagnosing non-small-cell lung cancer. One combination may be miR-17-5p, cytokeratin 19 fragment, carcinoembryonic antigen, and squamous cell carcinoma antigen, which in one study showed an area under the receiver operating characteristic curve of 0.860 during training and 0.844 during validation [119].

Breast cancer, which affects the mammary gland epithelium, is the most common malignancy affecting women, and it can rapidly metastasize to the lymph and blood [120]. Since early detection can substantially improve prognosis, its diagnosis based on liquid biopsy has received much attention. High-throughput sequencing of small RNAs in EVs from nine breast cancer cell lines indicated a different profile between this cancer and other types of cancer [121], suggesting the diagnostic potential of small RNAs. For example, levels of miR-1246 and miR-21 in plasma EVs are significantly higher in breast cancer patients than in controls [122], and levels of miR-233-3p in EVs have been associated with the degree of malignancy [123]. In addition to small RNAs, several proteins from EVs show diagnostic potential, such as HER2, CD47, DEL-1, and EpCAM, all of which are present at higher levels in patients than in controls [124, 125]. Beyond simply measuring protein levels to diagnose cancer, it may be possible to exploit changes in their posttranslational modifications. A study based on label-free quantitative phosphoproteomics identified several proteins in plasma EVs whose phosphorylation was increased in association with breast cancer, including cGMP-dependent protein kinase 1, nuclear transcription factor, X box-binding protein 1, Ral GTPase-activating protein subunit alpha-2, and tight junction protein 2 [11].

Pancreatic cancer is a highly malignant tumor. Its early symptoms are not typical and obvious, so early diagnosis is key to a better prognosis. Several miRNAs in serum EVs are upregulated in pancreatic cancer, including miR-17-5p, miR-21, miR-1246, miR-4644, miR-3976, and miR-4306 [126]. Levels of glypican-1, a cell surface proteoglycan, are enriched in exosomes from patients [43, 44], as are levels of serum exosomal proteins CD44v6, Tspan8, EpCAM, MET, and CD104 [127]. Diagnosis may become more specific and sensitive by assaying levels of the proteins GPC1 and CD82 in EVs together with levels of the well-established serum protein carbohydrate antigen 19-9 [128]. Colorectal cancer becomes obviously symptomatic only after it has reached an advanced stage, and its diagnosis depends on colonoscopy. Less invasive methods of early diagnosis may substantially improve prognosis. Seven miRNAs in serum EVs are upregulated in patients and may therefore be useful as biomarkers: let-7a, miR-1229, miR-1246, miR-150, miR-21, miR-223, and miR-23a [129]. At least one protein from serum EVs, CD147, may be useful as a biomarker [130].

Ovarian cancer, the fifth most frequent cancer affecting women, cannot be effectively screened because appropriate biomarkers are lacking. Studies suggest that several EV-derived miRNAs may be useful for this purpose, including miR-21, miR-141, miR-200a, miR-200b, miR-200c, miR-203, miR-205, and miR-214 [131]. At least one protein derived from plasma EVs, claudin-4, may be useful for diagnosis [132]. Serum-derived EVs from patients with nasopharyngeal carcinoma, a malignancy associated with Epstein-Barr virus (EBV) infection, have been shown to contain EBV components, including latent membrane protein-1 (LMP-1), BamHI-A rightward frame 1 (BARF1), and nucleic acids [133]. Such EV-derived proteins may serve as a more accurate alternative for diagnosis compared with anti-IgA/IgG and anti-VCA tests [134]. Galectin-9 is abundant in nasopharyngeal carcinoma cells infected by EBV, and EVs from the plasma of patients have also been shown to contain galectin-9 [135]. Several EBV BART miRNAs, including BART7-3p, BART9-3p, BART17, and especially BART13-3p, are more abundant in exosomes from the serum of patients than in exosomes from the serum of healthy controls [136–138]. In fact, exosomal miR-BART13-3p shows higher diagnostic specificity and sensitivity than traditional methods [136]. Therefore, these EV-derived miRNAs may be useful as a screening tool for diagnosing nasopharyngeal carcinoma.

#### 5.1.2. Blood-Based Liquid Biopsy of Noncancer Diseases

Blood-derived EVs have shown potential for the diagnosis of neurodegenerative diseases, including Alzheimer's disease, amyotrophic lateral sclerosis, Parkinson's disease, and Huntington's disease [139]. The calmodulin-binding protein neurogranin, expressed primarily in the brain, is downregulated in plasma EVs from Alzheimer's patients, and its levels correlate with those of cognitive biomarkers [140]. Identifying reliable biomarkers of Parkinson's disease may be particularly beneficial because treatments exist that can alter the disease and improve prognosis [141]. Levels of *α*-synuclein in plasma EVs are significantly higher in patients with early-stage Parkinson's disease than in healthy controls, and higher levels appear to be associated with a greater risk of progression of motor symptoms [142]. Thus, *α*-synuclein may be a useful diagnostic and prognostic biomarker. Blood-derived EVs have shown potential for the early detection of cardiovascular diseases. Levels of the long noncoding RNA SOCS2-AS1 in plasma EVs are higher in patients with coronary artery disease than in controls, and the RNA itself may help protect against the disease [143]. Levels of miR-1915-3p, miR-4507, and miR-3656 in serum EVs are significantly lower in patients who have suffered acute myocardial infarction than in controls, suggesting that those RNAs may help predict such events, which are a major cause of mortality worldwide [144]. MicroRNAs in EVs may also serve as biomarkers of cardiovascular diseases, such as exosomal miR-183 for predicting myocardial infarction [145].

### 5.2. Urine

#### 5.2.1. Urine-Based Liquid Biopsy of Cancer

Urine can be sampled noninvasively. Its contained EVs have cargoes that may help diagnose prostate cancer and bladder cancer. The disease often does not cause obvious symptoms until later stages, when hematuria or pelvic pain may occur. Screening for the disease relies on a digital rectal exam (DRE) and an assay of prostate-specific antigen (PSA). There is a worldwide consensus that PSA screening for prostate cancer often leads to overdiagnosis, leading researchers to search for better biomarkers [146]. High-throughput mass spectrometry of lipids in urinary EVs identified nine lipid species whose levels differed significantly between patients and controls [65]. These biomarkers may provide diagnostic information. Similarly, proteins from urinary EVs may help diagnose prostate cancer, such as fatty acid-binding protein 5 [147] as well as TGM4, ADSV, Flotilin2, and PARK7 [148, 149]. EV-derived RNAs may also aid in the diagnosis, such as miR-196a-5p and miR-501-3p [150]. In fact, levels of miRNA miR-2909 in urinary EVs correlate with the severity of prostate cancer [151]. Similarly, blood-derived EVs have also been investigated as noninvasive biomarkers for prostate cancer. Recently, Li et al. discovered that plasma exosomal miR-125a-5p and miR-141-5p performed well as diagnostic biomarkers of prostate cancer [152]. Proteomic analysis of serum exosomes also identified seven proteins present in prostate cancer patients but not in healthy individuals [153]. Further studies that compare the diagnostic accuracy of EVs isolated from blood or urine are still needed.

Bladder cancer is the second most frequent urinary tract cancer, affecting nearly 2 million people globally [154]. As in prostate cancer, the symptoms of bladder cancer are hematuria and pelvic pain, and patients usually become symptomatic when the disease is already in the intermediate or advanced stages. Bladder cancer appears to be associated not only with higher levels of urinary EVs [155] but also with higher levels of certain proteins within those EVs, such as alpha-1-antitrypsin and H2B1K, whose levels correlate with the grade of urothelial carcinoma [156]. Bladder cancer is also associated with higher levels of the proteins TALDO1, EPS8, and CEAM5 in EVs [157]. Similarly, several RNAs in urinary EVs may have diagnostic usefulness [158]: for example, the panel of long noncoding RNAs MALAT1, PCAT-1, and SPRY4-IT1 showed an area under the receiver operating characteristic curve of 0.813 for diagnosing bladder cancer [159]. A recent study showed that as biomarkers in the detection of bladder cancer, exosomes in serum and urine of patients increased with the invasiveness of tumors [160]. However, diagnostic sensitivity was higher when tumor-derived exosomes were isolated from urine than from serum, consistent with the report that urine was a suitable source of EVs for detecting kidney, bladder, and prostate disorders [161]. Another carcinoma that could potentially be diagnosed by EVs isolated from urine is renal cell carcinoma (RCC). In patients with RCC, exosomal proteins such as matrix metalloproteinase 9, ceruloplasmin, podocalyxin, Dickkopf-related protein 4, and carbonic anhydrase IX are upregulated, but AQP1, extracellular matrix metalloproteinase inducer, neprilysin, dipeptidase-1, and syntenin-1n are downregulated [162]. It may be similar to EV-derived miRNAs as biomarkers, such as miR-126-3p, miR-449a, and miR-34b-5p. The combination of miR-126-3p and miR-34b-5p can discriminate patients with small renal masses from healthy individuals, and the combination of miR-126-3p and miR-486-5p can discriminate benign lesions from clear cell RCC [163].

#### 5.2.2. Urine-Based Liquid Biopsy of Noncancer Diseases

Since renal cells release EVs, analysis of urinary EVs may provide insights into the state of the health of the kidney. In fact, levels of the kidney proteins gelatinase and ceruloplasmin in urinary exosomes appear to provide a better index of kidney health in patients with diabetic nephropathy than analysis of total urinary protein, potentially allowing better treatment and management to avoid progression to end-stage renal disease [164, 165]. Levels of the multiligand endocytic receptor C-megalin increase in urinary EVs as diabetic nephropathy progresses [166], while epithelium-specific transcription factor Elf3 is detectable in urinary EVs from patients with diabetic nephropathy but not in EVs from controls [167]. Thus, Elf3 may be a useful marker of irreversible podocyte injury in the early stages of diabetic nephropathy. Another potential marker for the early disease may be Wilm's tumor-1 protein in urinary EVs [168, 169], and exosomal levels of the mRNA encoding this protein may correlate with glomerular damage, providing diagnostic and prognostic value [170]. Autoantibody-induced renal damage can lead to lupus nephritis, one of the most common and severe forms of secondary glomerulonephritis. Flare-ups of this disease have been associated with downregulation of the miRNAs let-7a and miR-21 in urinary EVs [171]. Multimarker panels may provide more specific and sensitive diagnoses than single markers: the biomarker panel miR-21, miR-150, and miR-29c can detect early fibrosis formation in lupus nephritis as well as predict disease progression [172]. Urinary EVs may also be useful for diagnosing Alzheimer's disease: levels of A*β*1-42 and P-S396-tau are higher in patients than in controls [173]. In fact, EVs may contribute to the disease by shuttling toxic amyloid-beta and hyperphosphorylated tau between cells.

### 5.3. Saliva

#### 5.3.1. Saliva-Based Liquid Biopsy of Cancer

As one of the most prevalent cancers worldwide, oral squamous cell carcinoma (OSCC) accounts for around 90% of oral malignant tumors [174]. Because of its unapparent symptoms at the early stage, patients could easily and unconsciously miss the best diagnostic period; thus, sensitive biomarkers are under urgent demand. Cancer cells can secrete EVs into saliva, and this secretion appears to be related to tumor invasion or metastasis; thus, salivary EVs appear to differ between OSCC patients and healthy individuals, such as in levels of the proteins CD63, CD81, and CD9 [175]. Numerous miRNAs in salivary EVs have also been associated with OSCC, including miR-517b-3p, miR-302b-3p, miR-412-3p, and miR-512-3p [176], as well as miR-24-3p, whose target gene is Period 1 and which gave an area under the receiver operating characteristic curve of 0.738 for diagnosing OSCC [177].

Most strikingly, the studies of salivary EV biomarkers have developed beyond oral cancer. The potential of salivary EVs to detect cancer early may extend to lung cancer. Several studies have reproducibly found various proteins whose levels in salivary EVs differ between lung cancer patients and controls. Sun et al. have been working to explore the utility of salivary EV proteins for lung cancer detection. In 2016, this research group proposed shotgun proteomic analysis illustrating 12 salivary EV proteins that could only be discovered in lung cancer patients [178]. In 2017, they compared salivary and serum exosomal proteomes of lung cancer by LC/MS, and 11 potential proteins were demonstrated in both body fluids, which indicated that both biofluids contain valuable biomarkers [113]. In 2018, they isolated exosomes and microvesicles in human saliva from lung cancer patients and normal subjects. In particular, they identified 5 exosomal proteins and 9 microvesicle proteins, including BPIFA1, CRNN, MUC5B, and IQGAP, as lung-related proteins [179]. Salivary EVs have also been investigated for their diagnostic potential in other cancers. Levels of Melan-A RNA within salivary EVs are upregulated in patients with melanoma [180], while exosomal levels of miR-1246 and miR-4644 may have diagnostic potential in pancreatobiliary tract cancers [181]. Levels of several DNA molecules in salivary EVs appear to be upregulated in pancreatic cancer: Apbb1ip, Daf2, Foxp1, Incenp, Aspn, BC031781, and Gng2 [182]. Similarly, levels of several miRNAs in salivary EVs are upregulated in head and neck squamous cell carcinoma: miR-486-5p, miR-10b-5p, and miR-486-3p [183, 184].

#### 5.3.2. Saliva-Based Liquid Biopsy of Noncancer Diseases

Oral lichen planus, a chronic inflammatory disorder of the oral mucosa, can become malignant. Levels of miR-4484 in salivary exosomes may help diagnose this condition [185]. Periodontitis, which causes alveolar bone resorption [186], has been associated with elevated exosomal levels of mRNA encoding PD-L1 [187]. Sjögren's syndrome, a common long-term autoimmune disease, has been associated with elevated levels of proteins of saliva EVs involved in innate immunity, cell signaling, and wound repair [188]. The disease has also been associated with upregulation of exosomal levels of guanine nucleotide-binding protein subunit alpha-13, adipocyte plasma membrane-associated protein, WD repeat-containing protein 1, lymphocyte-specific protein 1, and tyrosine-protein phosphatase nonreceptor type substrate 1 [189]. Inflammatory bowel disease has been linked to altered levels of proteasome subunit alpha type-7 in salivary EVs [190]. Finally, although aging should perhaps not be considered a disease, age-dependent changes in immune and inflammatory responses, as well as apoptosis of salivary gland cells, have been associated with levels of miR-24-3p in salivary EVs [191].

## 6. Perspective and Conclusion

Liquid biopsy has become a hot topic in molecular diagnostics. A growing number of studies have highlighted the potential diagnostic value of EVs. Recently, EVs are considered an attractive liquid biopsy tool as they can sensitively reflect an individual's health status. Although EV-based liquid biopsy has great potential for clinical application, obvious limitations exist. First, different body fluids have different characteristics such as density and viscosity, as well as specific subpopulations of EVs. Hence, EVs isolated from different biofluids may differ in diagnostic accuracy for the same disease. For example, in melanoma patients, lymphatic exudate contains more cancer-derived EVs than plasma, and lymph vessels were shown to be the major route of EV transport from tumors into the circulation [192, 193]. In lung cancer, tumor-draining pulmonary venous blood contains more cancer-derived EVs than peripheral blood [194]. Further studies are needed to develop standard protocols for sampling different biofluids and isolating EVs from them, as well as for establishing the diagnostic accuracy of those EVs. Second, clinical studies of EV-derived biomarkers typically involve small samples rather than large, longitudinal studies. So large sample trials are needed in the future to establish robust evidence. Third, many EV-derived biomarkers lack specificity, so the same biomarkers can be present in multiple diseases. For example, HER3 is upregulated in breast and lung cancers, while CD24 is abundant in ovarian and breast cancers. Therefore, an accurate diagnosis of a given disease may require detection of the combination of different biomarkers.

In conclusion, this review highlighted the current status of EV-based biomarkers in liquid biopsy and described their biogenesis, contents, and isolation from different biofluids. There is still a long road ahead to the clinical usage of EV-based liquid biopsy, but its potential diagnostic potential still excites and drives scientists to further research on it.

## Figures and Tables

**Table 1:** Examples of potential biomarkers from extracellular vesicles.

Body fluid	Disease	Proteins and fatty acids	Nucleic acids	References
Blood	Non-small-cell lung cancer	EGFR, NY-ESO-1, PLAP, EpCAM, Alix, fibronectin, AHSG, and ECM1	GAS5, miR-17-5p, cytokeratin 19 fragment, carcinoembryonic antigen, and squamous cell carcinoma antigen	117-121
	Breast cancer	HER2, CD47, DEL-1, EpCAM, PKG1, NFX1, RALGAPA2, TJP2	miR-1246, miR-21, miR-233-3p	11, 124-127
	Pancreatic cancer	Glypican-1, CD44v6, Tspan8, GPC1, EpCAM, MET, CD104, CD82, LysoPC 22:0	miR-17-5p, miR-21, miR-1246, miR-4644, miR-3976, miR-4306	45, 46, 128-130
	Colorectal cancer	CD147	let-7a, miR-1229, miR-1246, miR-150, miR-21, miR-223, miR-23a	131,132
	Ovarian cancer	Claudin-4, cholesterol ester, zymosterol	miR-21, miR-141, miR-200a, miR-200b, miR-200c, miR-203, miR-205, miR-214	65, 133, 134
	Nasopharyngeal carcinoma	Galectin-9	BART7-3p, BART9-3p, BART17, BART13-3p	137-140
	Alzheimer's disease	Neurogranin	Unknown	142
	Parkinson's disease	*α*-Synuclein	Unknown	144
	Coronary artery disease	Unknown	SOCS2-AS1	145
	Acute myocardial infarction	Unknown	miR-1915-3p, miR-4507, miR-3656, miR-183	146

Urine	Prostate cancer	FABP5, TGM4, ADSV, Flotilin2, PARK7, phosphatidylserine (18:1/18:1), lactosylceramide (d18:1/16:0)	miR-196a-5p, miR-501-3p, miR-2909	67, 149-153
	Bladder cancer	*α*1-antitrypsin, H2B1K, TALDO1, EPS8, CEAM5	MALAT1, PCAT-1, SPRY4-IT1	158-159, 161
	Renal cell carcinoma	CP, PODXL, CD10, MMP9, EMMPRIN, CAIX, DPEP1, DKK4, Synten-in1, and AQP1	miR-126-3p, miR-449a, miR-34b-5p	164-165
	Diabetic nephropathy	C-megalin, Elf3, WT1	mRNA WT1	168-171
	Lupus nephritis	Unknown	let-7a, miR-21, miR-21, miR-150, miR-29c	173-174
	Alzheimer's disease	A*β*1-42, P-S396-tau	Unknown	175

Saliva	Oral squamous cell carcinoma	CD63, CD81, CD9	miR-517b-3p, miR-302b-3p, miR-412-3p, miR-512-3p, miR-24-3p	177-179
	Lung cancer	BPIFA1, CRNN, MUC5B, IQGAP	Unknown	181
	Melanoma	Unknown	Melan-A RNA	182
	Pancreatobiliary tract cancer	Unknown	miR-1246, miR-4644	183
	Pancreatic cancer	Unknown	Apbb1ip, Daf2, Foxp1, Incenp, Aspn, BC031781, Gng2 mRNA	184
	Head and neck squamous cell carcinoma	Unknown	miR-486-5p, miR-10b-5p, miR-486-3p	185-186
	Oral lichen planus	Unknown	miR-4484	187
	Periodontitis	Unknown	PD-L1 mRNA	188
	Sjögren's syndrome	GNA13, APMAP, WDR1, LSP1, SIRPA	Unknown	191
	Inflammatory bowel disease	PSMA7	Unknown	192
	Aging process	Unknown	miR-24-3p	193

## Data Availability

No data were used to support this study.
